# A Role for the PPAR**γ** in Cancer Therapy

**DOI:** 10.1155/2008/314974

**Published:** 2008-05-28

**Authors:** Moray J. Campbell, Carsten Carlberg, H. Phillip Koeffler

**Affiliations:** ^1^Department of Pharmacology and Therapeutics, Roswell Park Cancer Institute, Elm and Carlton Streets, Buffalo, NY 14263, USA; ^2^Department of Biosciences, University of Kuopio, 70211 Kuopio, Finland; ^3^Life Sciences Research Unit, University of Luxembourg, 1511 Luxembourg, Luxembourg; ^4^Division of Hematology/Oncology, Cedars-Sinai Medical Center, Los Angeles School of Medicine, University of California, 8700 Beverly Boulevard, Los Angeles, CA 90048, USA

## Abstract

In 1997, the first published reports highlighted PPAR*γ* as a novel cancer therapeutic target regulating differentiation of cancer cells. A subsequent flurry of papers described these activities more widely and fuelled further enthusiasm for differentiation therapy, as the ligands for the PPAR*γ* were seen as well tolerated and in several cases well-established in other therapeutic contexts. This initial enthusiasm and promise was somewhat tempered by contradictory findings in several murine cancer models and equivocal trial findings. As more understanding has emerged in recent years, a renaissance has occurred in targeting PPAR*γ* within the context of either chemoprevention or chemotherapy. This clarity has arisen in part through a clearer understanding of PPAR*γ* biology, how the receptor interacts with other proteins and signaling events, and the mechanisms that modulate its transcriptional actions. Equally greater translational understanding of this target has arisen from a clearer understanding of in vivo murine cancer models. Clinical exploitation will most likely require precise and quantifiable description of PPAR*γ* actions, and resolution of which targets are the most beneficial to target combined with an understanding of the mechanisms that limits its anticancer effectiveness.

## 1. CURRENT UNDERSTANDING OF PPAR*γ* BIOLOGY

### 1.1. PPAR*γ* is a transcription factor

The human PPAR*γ* was cloned in 1994 and subsequently two murine isoforms were identified in mouse: gamma-1 and gamma-2, resulting from the use of different initiator methionines [1, 2]. Subsequently, at least three isoforms have been identified in humans with common expression in adipocytes and the large intestine and more restricted isoform expression in other tissues [3]. PPAR*γ* plays a key role in energy metabolism and differentiation (reviewed in [4–7]); and reflecting this, the murine *Ppar* *γ* ^−/−^ is embryonically lethal, and if rescued, the animal lacks normal adipocytes [8].

PPAR*γ* is a phylogenetic member of subfamily 1 the nuclear receptor (NR) superfamily and shares a number of generic mechanistic features in common with other subgroup members, including the retinoic acid receptors (RARs), vitamin D receptor (VDR), farnesoid X receptor (FXR), and liver X receptors (LXRs). These receptors are most commonly located in the nucleus and heterodimerize with one of three retinoid X receptor (RXR) subtypes, to bind specific response elements in target gene regulatory regions. Crystallization studies of PPAR*γ* bound with RXR*α* proved pivotal for deciphering the basis for heterodimerization with RXR for multiple NRs [9]. The presence of ligand changes the receptor confirmation and also influences choice of association with either coactivator (CoA) or corepressor (CoR) complexes. In the absence of ligand, NR heterodimers are contained within multimeric complexes (∼2.0 MDa) containing CoRs (e.g., NCOR1) [10]. Also, within these complexes is a range of enzymes, which act to modify the posttranslational status of histone tails and maintain a locally closed repressive chromatin environment, for example, histone deacetylases (HDAC), such as HDAC3 and SIRT1 [10–15].

Ligand activation shifts receptor conformation and distribution to enhance interaction with CoA complexes. A large number of interacting CoA proteins have been described, which can be divided into multiple families including the NCOA/SRC family and members of the large bridging mediator complex including PPAR*γ* binding protein (PBP/MED1) complex [16, 17]. Through the latter, the NR receptor complex links to the cointegrators CBP/p300 and basal transcriptional machinery. For example, PPAR*γ* is known to associate with proteins, such as SRC-1, PGC1-*α*, CARM1, and a battery of histone modifying enzymes, such as histone acetyltransferases (HAT), which together initiate and promote transactivation [18–22].

The complex choreography of these events is a very active area of research, being at a crossroads of several important areas in contemporary biology, such as multimeric protein complex assembly and chromatin remodeling. Transcription involves cyclical rounds of promoter-specific complex assembly, gene transactivation, complex disassembly, and proteosome-mediated receptor degradation [23–25].

### 1.2. Newly characterized and unique features of PPAR*γ*

Outside of these general characteristics, uncertainty and ambiguity remain in constructing a predicative schema for understanding PPAR*γ* function and signaling in cancer biology. Some of the uncertainties arise due to a number of structural and regulatory variations of PPAR*γ* outside the core features of NRs, thereby leading to apparently pleiotropic actions. Compounding these difficulties is the issue of studying PPAR*γ* signaling in cancer biology, which is intrinsically an unstable and evolving disease environment.

**Figure 1:**
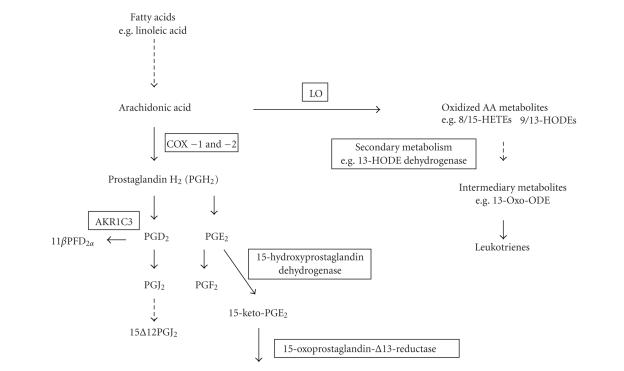
Generation of natural PPAR*γ* ligands (solid arrows = direct conversion, broken arrows = multiple step conversion. Metabolites are indicated and some of the key regulating enzymes are shown in boxes).

By contrast to a high-affinity receptor, such as estrogen receptor *α* (ER*α*), the members of the subfamily 1 of the NR superfamily are typified by their large ligand-binding domain and may therefore accept different ligands. The PPAR*γ* ligand-binding pocket has a volume of more than 1400 Å^3^ and therefore can bind a wide range of different lipophilic molecules (see Figure 1). As shown in Figure 1, free fatty acids are metabolized to arachidonic acid, and then through either lipooxygenase (LO) or cyclooxygenase (COX) activities to give rise to a range of natural ligands for PPAR*γ*. Many of these reactions are tightly controlled such that a ligand metabolite is enzymatically generated and cleared.

Circulating and cellular fatty acids give rise to the majority of the natural ligands for PPAR*γ*; therefore, the PPARs in general and PPAR*γ* specifically form a sensing mechanism to maintain homeostasis in changing physiological circumstances such as feeding and exercise. This capacity, as discussed later, is implicated in a range of disease settings including cancer. The omega 6 fatty acid, linoleic acid, is highly inflammatory and therefore carefully controlled *in vivo*. It is a PPAR*γ* ligand and, through subsequent desaturase and elongase activities, is metabolized to arachidonic acid. A wide range of natural ligands for PPAR*γ* is subsequently derived through arachidonic acid metabolism. LO activity (e.g., arachidonate 5-LO and 15-LO) generates oxidized lipids which act as PPAR*γ* ligands, such as 8 (S)-hydroxyeicosatetraenoic acid (8-(S)-HETE), 15-(S)-HETE, 9-hydroxy-10,12-octadecadienoicacid (9HODE), and 13-HODE. Subsequent dehydrogenase activity, for example, of 13-HODE by 13-HODE dehydrogenase, can result in a further series of PPAR*γ* ligands prior to their subsequent conversion to leuktrienes [26–28].

In parallel, arachidonic acid can be metabolized through cyclooxygenase activity (through COX-1 and -2) to prostaglandins such as PGH_2_ and subsequently PGD_2_, PGE_2_, PGF_2_, and PGI_2_. These compounds exert a diverse range of cellular actions, but key metabolites in these cascades appear to exert potent PPAR*γ* activation. PGD_2_, the product of prostaglandin D synthase (encoded by *PGDS*), is able to undergo nonenzymatic degradation to a J series prostaglandin, 15-deoxy-^12,14^-prostaglandin J_2_ (15d-PGJ_2_), which is a potent PPAR*γ* ligand [26, 29–33]. Similarly, metabolites of PGE_2_ can activate PPAR*γ*, and their generation is controlled during differentiation, for example, of adipocytes [34]. Many of these reactions appear to be regulated through classical feedback loops, thus, the regulation of arachidonic acid metabolism to provide prostaglandins and leukotrienes is regulated at multiples levels by the actions of PPAR*γ*, for example, regulation of LOs and of COX-2 activity and several of the downstream enzymes [26, 29–35].

The discovery of synthetic ligands for this receptor has been driven by the identification of a number of significant disease settings, in which PPAR*γ* signaling is implicated (inflammation, metabolic disorders, and cancer). A goal of this research is the identification of novel pharmacological compound that display gene- and cell-selective actions [36]. The diversity of cell function, and presumably the relatively large ligand-binding pocket, has encouraged investigators to undertake rational screening approaches to identify a diverse panel of ligands [31, 37–51]. Indeed, novel selective compounds frequently display differential ligand-binding pocket docking sites. Implicit, within these discoveries is that the subtly different induced receptor conformations allow for the different spatiotemporal associations of CoA and ancillary proteins thereby deriving target gene specificity [40, 41, 52–55]. Thiazolidinediones (TZDs) were the first synthetic compounds investigated as PPAR*γ* ligands [56]; this class also includes rosiglitazone, pioglitazone, and troglitazone. The latter caused a severe idiosyncratic liver problem and thus has been discontinued. The TZDs have proven to be a breakthrough in the therapy of type II diabetes because they decrease insulin resistance by promoting glucose uptake, mitochondrial biogenesis and fatty acid absorption by increasingly differentiated adipocytes (reviewed in [57]).

This focus at the level of the PPAR*γ* ligand may be too exclusive. For example, the RXR member of this complex can also bind simultaneously with its ligand, which can result in enhanced transcriptional activity (6). Perhaps more importantly, the receptor structure allows it to influence both the basal and regulated transcription levels of target genes independent of ligand. That is, the unliganded structure of PPAR*γ* also exposes a number of critical amino acids on helix 12 that allows CoA binding and may explain the high basal expression levels of PPAR*γ* target genes in the absence of ligand. In this regard, PPAR*γ* most closely resembles another xenobiotic metabolizing NR, constitutive androstane receptor (CAR) [58]. These findings may also suggest that the expression of CoA and CoR proteins are actually more important for regulating gene targets than either the levels or specificity of ligands.

The biology of PPAR*γ* is intimately associated with that of the PGC-1*α* CoA and a number of other cofactors. The actions of these proteins have most clearly been described in well-established PPAR*γ* systems, such as adipocyte differentiation and regulation of energy metabolism. The Pgc-1*α* murine knockout displays abnormal metabolic rates, temperature fluctuations, and a lethal cardiac defect [59, 60]. Reflecting its importance for regulating PPAR*γ* function, levels of PGC-1*α* are tightly regulated by ubiquitination [61].

PPAR*γ* receptor activity is also regulated by a cohort of posttranslational mechanisms, such as small ubiquitin-related modifier (SUMO) process. Sumoylation of the ligand-binding domain, in the presence of ligand, prevents the release and subsequent ubiquitination of NCOR1, and therefore sustains the repressive action, leading to the so-called ligand-dependent transrepression [62, 63]. This process is antagonized, by the removal of the SUMO modification by the SUSP-1 enzyme [64] thus establishing a dynamic level of regulation to modify the actual impact of ligand. Furthermore, PPAR*γ* is serine phosphorylated, for example, in response to MAPK signaling leading to nuclear export and attenuation of transcriptional ability [65–67]. By contrast, PBP/MED1 is regulated at multiple sites by phosphorylation to enhance signaling by PPAR*γ* [68].

To place the expression and regulation of PPAR*γ* within the broader context of NR biology, several scientists have proposed and utilized system level approaches to dissect NR function including PPAR*γ*. One of the most significant examples of this approach has been the spatiotemporal profiling of all 49 murine NRs in multiple tissues at different time points during the circadian rhythm [69, 70]. These approaches have revealed a number of provocative findings. In terms of tissue expression, *Ppar* *γ* most closely follows *Lxr* *α* and *Gr*, and forms a triumvirate that is intimately implicated in the control of inflammation. The expression of PPAR*γ* was shown also to follow circadian rhythm expression in white adipose tissue and the liver, but not other tissues [69, 70]. Similarly, others have shown that *Pgc-1* *α* follows a circadian rhythm in the liver and skeletal muscle of mice [20], and it cooperates with other NRs to regulate additional members of the clock family.

### 1.3. Transcriptional targets of PPAR*γ*

One approach to defining PPAR*γ* specificity has been to describe the cohort of target genes regulated by its actions; generally, these studies involve microarray studies in a range of cell types including adipocytes [71] and macrophages [72]. Commonly, a range of gene targets has been identified associated with metabolism and transport of lipids, including lipoprotein lipase, fatty acid binding, and transport proteins and acyl-CoA synthase. Similar approaches have been used to study the impact of PPAR*γ* signaling on proliferation and differentiation. For example, in chondorosarcoma and ovarian cancer cells, PPAR*γ* actions were associated with changes in the ratio of BAX to BCL-2, induction of programmed cell death [73], and upregulation of cyclin-dependent kinase inhibitors (CDKIs), such as *CDKN1A* (encodes p21^(waf1/cip1)^) [74]. In MCF-7 breast cancer cells PPAR*γ* upregulated a similar spectrum of CDKIs [75]. A number of studies have identified the IGF axis as a target of PPAR*γ* signaling. For example, in bone marrow cells [76], and *in silico* and in vitro studies have characterized a range of PPAR response elements (PPREs) in several insulin-like growth factor binding protein (*IGFBPs*) genes [77]. Other scientists have attempted to increase the accuracy of gene target identification by using selective ligands, for example, in colorectal cells, and identified gene targets associated with mitotic restraint and cell adhesion [78–82]. Complimentary approaches have utilized adenoviral transfection of receptor subtypes to identify differentially expressed genes, confirmed with chromatin immunoprecipitation (ChIP) approaches [83].

The accurate prediction of target genes is compounded by the highly integrated nature of PPAR*γ* signaling with other NR family members. For example, its activities are mutually antagonized with ER*α* signaling, and appear to be cooperative with both VDR and RAR, in part by increased retinol synthesis [84–86]. To investigate this apparent transcriptome plasticity will require the integrations of *in silico* response element identification protocols combined with ChIP-sequencing approaches to establish specificity and redundancy; comparable approaches have been undertaken for ER*α* [87]. Building towards this goal, we have undertaken a meta-analyses of PPRE sequences to generate an algorithm to predict PPAR subtype binding and screened chromosome 19, as a test set, to identify and confirm a number of novel genes [88].

Together, these findings suggest that ligand is just one of a number of mechanisms to regulate receptor function. Other regulatory contributions are determined by PPAR*γ* expression level, isoform, posttranslational modification, location, crosstalk with functionally related receptors and cofactor expression. Together, these components combine with wider transcriptional programs, such as energy utilization, circadian rhythm, and the control of inflammation to drive and specify the timing of transcriptional outputs.

## 2. CONTROL OF SELF-RENEWING TISSUES

### 2.1. Common cancers and leukemia arise in self-renewing tissues

The weighted contribution of the underlying forces, acting at the levels of genes, chromosomes, signaling cascades and tissue organization, that drive cancer initiation and progression remain poorly understood. Historically, a paradigm of exclusive genetic causality was the basis for investigating cancer etiology and it identified certain key nodal points of cellular control, such as p53. In the postgenomic era, other strong penetrance genes have not been readily identified. The sporadic, multistage acquisition of a cancer phenotype requires disruption of multiple mechanisms of cellular restraint and tissue organization (reviewed in [89]). Reflecting a sporadic multifactorial cancer phenotype, the single greatest risk factor for most cancers is age, with the average age of onset of breast, prostate, and colon cancer in the sixth and seventh decades of life.

Further understanding of transformation processes has arisen through appreciation of the diverse cell types present at the sites of high-profile malignances. Epithelial linings of the prostate and mammary glands, the gastrointestinal tract and hematological systems all typify self-renewing tissues containing stem cell populations [90–94]. These cells give rise to committed progenitors, and in turn the multiple-cell lineages required for tissue function. Stem cells are relatively rare and long-lived, but frequently quiescent. They are uniquely able to undergo asymmetric division, to give rise to both other stem cells and transiently amplifying populations of progenitor cells, that in turn give rise to the differentiated cell types. The differentiated epithelial cells are functional but short-lived and lost through programmed cell death processes, to be replaced by newly differentiated transiently amplifying cells. Cellular control of the intricate balance of the processes of division, differentiation, and programmed cell death include common roles for Wnt, Hedgehog, and other developmental signal transduction processes. Convergent targets for these signals include key regulators of cellular proliferation, such as Myc and p21^(waf1/cip1)^.

As a result of their long life cycle and high proliferative capacity, stem cells, rather than their short-lived terminally differentiated daughter cells, are the candidates for transformation. However, a range of mechanisms is in place to maintain stem cell genomic integrity, perhaps including retention of the so-called “immortal” DNA strand and enhanced protection mechanisms [95–103]. These controls notwithstanding, the transformation of stem cells has given rise to the concept of cancer stem cells. Such cancer stem cells are well established in leukemia and accumulating evidence supports the presence of these cells in prostate, breast, and colon cancers [104–108].

### 2.2. Restoration of controlled self-renewal as a therapeutic goal

Members of the NR superfamily play a number of well-established roles in the control of self-renewal and the process of normal differentiation. For example, the AR and ER*α* receptors play pivotal roles in prostate and breast tissue development and maintenance. Distortion of some of these actions is, in turn, central to the development of cancer in these tissues and is targeted therapeutically though antagonism, either completely in the case of the AR, or selectively in the case of the ER*α*. Agonism of other receptors has been pursued to induce differentiation and inhibit proliferation of cancer cells. The best example of this paradigm is the induction of remission of patients with acute promyelocytic leukemia using the RAR ligand, all-*trans* retinoic acid, and also to prevent recurrence of head and neck cancers.

As a consequence of the induced terminal differentiation of normal preadipocytes by ligands for PPAR*γ* [1, 2], investigators were encouraged to use TZDs to attempt to induce differentiation of human liposarcoma cells *in vivo* [109]. Successes in vitro encouraged these same physician-scientists to give troglitazone to a series of patients with liposarcoma, which resulted in a retardation of growth and induction of differentiation of these tumor cells. The long-term effect of TZD on liposarcomas requires further study; nevertheless, these pioneer studies spurred the examination of the effect of TZDs on a number of cancers both in vitro and *in vivo* in colon, breast, prostate, myeloid leukemia, neuroblastoma, glioblastoma, lymphoma, lung, cervical, bladder, head and neck, esophageal, gastric, pancreatic, and choriocarcinoma cancers [21, 81, 110–140]. The multiple findings from studies illustrate the promise and failings of targeted therapies toward PPAR*γ* to restore mitotic restraint and induce differentiation.

## 3. PPAR*γ* SIGNALING IN CANCER

### 3.1. Colon cancer

To establish a role for PPAR*γ* to protect against the development of colon cancer, investigators have used a range of *in vivo* and in vitro approaches. In murine models, the expression of *Ppar* *γ* has been manipulated in either an environmental or a genetic background that displays enhanced susceptibility to colonic cancer. For example, mice with heterozygous germ-line deletions of *Ppar* *γ* have an increased proclivity to develop *N*-methyl-*N*-nitrosourea carcinogen-induced colon cancer compared with wild-type mice, supporting a growth inhibitory role for *Ppar* *γ*. Significantly, troglitazone reduced the tumor incidence in wild-type but not heterozygote mice [122]. By contrast, other scientists have utilized the well-established APC_*min*_ model of colon cancer with apparently contradictory findings. These mice have a germ-line mutation of the APC gene resulting in deregulated *β*-catenin signaling, and a very significantly increased frequency of small and large intestinal adenocarcinomas. Surprisingly, administration of TZD to APC_*min*_ mice resulted in increased frequency of colon cancers compared to control animals [141]. Subsequently, however, generation of the intestinal specific *Ppar* *γ* ^−/−^ and APC_*min*_ bigenic mouse demonstrated an unequivocal effect of *Ppar* *γ* to suppress tumor formation and suggests that significant off-target effects of TZD occur in mice, especially in the APC_*min*_ mouse colon cancer model [142]. Off-target effects of TZD generally appear to also have broad anticancer properties; therefore, the findings in this model appear quite unusual. For example, *Ppar* *γ* inactive analogs of TZD initiate the proteosomic degradation of *β*-catenin [143] and cyclin D1, as well as, interfering with BAX family member interactions to bring about apoptosis [144, 145]. Nevertheless, why APC_*min*_ mice receiving a TZD have more colon cancers still is not fully elucidated. APC_*min*_ mice have high levels of *Ppar* *γ* in the colonic cells and are inappropriately sequestrated by *β*-catenin to a unique set of gene targets [146]. Interestingly, PPAR*α* ligands inhibit polyp formation in the APC_*min*_ model [118] re-enforcing the concept that the TZD-driven enhanced tumor formation in the APC_*min*_ mouse is a model artifact, or at least not general phenomena.

In humans, multiple lines of evidence support an unequivocal function for PPAR*γ* signaling in colon cancer. Mutations of the receptor have been reported, although rare [147], and polymorphisms are functionally linked with an increased incidence of this cancer [148]. A range of natural and synthetic PPAR*γ* ligands inhibit proliferation, induce programmed cell death and exert prodifferentiation actions in vitro and *in vivo*, for example, when tested in human xenografts [149–151]. The potency of the ligand actions can be significantly enhanced further by combining the treatment with RXR ligands [124, 152]. Furthermore, this signaling capacity is integrated with the control of other proliferative signals, such as gastrin [153] (reviewed by [154]).

### 3.2. Breast cancer

The findings on breast cancer support the broad anticancer activities of PPAR*γ* signaling, and also reflect the studies in colon cancer. That is, generally in vitro and *in vivo* studies support a clear role for this receptor to suppress proliferation, induce differentiation and programmed cell death. In rodent models, the PPAR*γ* agonists block *N*-nitroso-*N*-methylurea-induced breast cancer in Sprague-Dawley rats [155] and DMBA-induced breast cancer in mice [114]. Similarly, *Ppar* *γ* ^+/−^ mice have a greater susceptibility to develop breast and ovarian cancers after their exposure to 7,12-dimethylbenz(*a*)anthracene [156].

By contrast, transgenic mice having a constitutively active PPAR*γ* in their breast tissue crossed with the MMTV-neu mouse model of breast cancer displayed accelerated kinetics of breast cancer development, although the authors noted that the tumors surprisingly were more secretory and differentiated in nature [157]. Similar to the APC_*min*_ model, this tumor model depends on deregulated Wnt activity, and the authors suggested that the effects may also reflect aberrant interplay between PPAR*γ* and Wnt signaling.

Human breast cancer cells express PPAR*γ* [158] and can be targeted, for example, with TZD, and a range of other PPAR*γ* ligands to induce differentiation and inhibition of cell growth both in vitro and in xenograft models, effects which can be enhanced by cotreatment with either retinoids, TGF*β* or TNF*α* [110, 111, 113, 114, 130, 158–163]. For example, PPAR*γ* ligands plus selective retinoid ligands converge on targets, such as RAR*β*, which is known to act as a tumor suppressor and is commonly silenced in malignancy [164]. Similarly, PPAR*γ* activation results in upregulation of E-cadherin and thereby redistribution of *β*-catenin [130]. Natural ligands, such as dietary fatty acids, change expression in syndecan-1 with an impact on cytoskeleton structure and the induction of apoptosis [165]. Furthermore, 15d-PGJ_2_ inhibits ER*α* signaling in a PPAR*γ*-independent manner by covalent modification of the receptor [166]. PPAR*γ* expression is a favorable prognostic factor [167] and associates with ER*α* positive disease [75]. A note of caution, however, phase II trials of TZDs in women with hormone refractory metastatic breast cancer were equivocal [168].

### 3.3. Prostate cancer

The biology of the prostate is intimately associated with the synthesis of prostaglandins, as suggested by the name. These growth regulatory factors are readily secreted by the gland [169] and give rise to the H and D series prostaglandins and 15d-PGJ_2_. Equally, the biology of the prostate is associated with the metabolism of fatty acids 15S-HETE [33]. Therefore, the prostate seems a tissue where PPAR*γ* may play a strong role in governing cell growth and differentiation. For example, signals derived from *PGDS* activity in the adjacent stroma, such as PGD_2_, activate PPAR*γ*, and control epithelial proliferation [170].

PPAR*γ* actions in prostate cancer cell lines [171] and primary cancer models [120] are well documented and include the induction of type II programmed cell death also known as autophagy [112]. These studies encouraged several groups to undertake clinical trials with PPAR*γ* ligands and disease stabilization was reported [115]. Again in this disease setting, PPAR*γ*-independent actions of TZDs were apparently identified, which were nonetheless potent anticancer signals [172, 173].

Set against these findings, the Evans team used a prostate cancer, the TRAMP model, to demonstrate that *Ppar* *γ* heterozygote mice have no change in disease progression compared to wild-type litter mates [174].

### 3.4. Leukemia and lymphoma

Previously, we showed that human myeloid and lymphoid leukemia cells express PPAR*α* and PPAR*γ*; ligands, such as troglitazone, inhibited their cell growth [139, 175]. This antiproliferative effect was markedly enhanced in the presence of various retinoids. Also, macrophages and myelomonocytic leukemic cells express abundant PPAR*γ* (73), and PPAR*γ* ligands can induce acute myelomonocytic leukemic cells (THP-1) to differentiate toward macrophages with an increased expression of the CD36 scavenger receptors, as well as other surface markers associated with differentiation including CD11b, CD14, and CD18 (73). Studies by others and us have also shown that PPAR*γ* ligands can inhibit growth and/or induce apoptosis of Hodgkin's disease [139] and multiple myeloma cells [176, 177]. The mechanism, by which PPAR*γ* ligand inhibits the proliferation of malignant hematopoietic cells, is not totally clear. Some of the antileukemic effects of PPAR*γ* may be independent of the PPAR*γ* receptor. Furthermore, we have found that a dual PPAR*α*/*γ* ligand (TZD18) has the ability to induce marked apoptosis and to inhibit growth of lymphoid leukemia cells [178]. In general, the effect of PPAR*γ* ligands on myeloid leukemic growth and differentiation is modest (74).

### 3.5. Mechanisms of resistance

**Figure 2:**
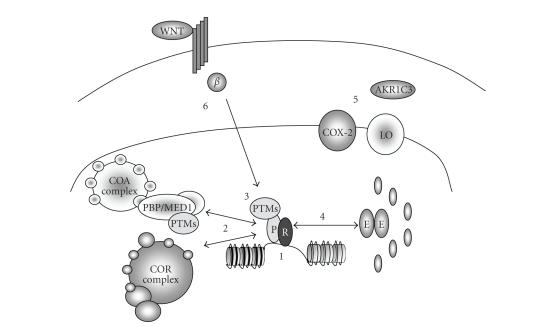
The actions of the PPAR*γ* to regulate target genes are highly choreographed, being influenced by many factors. This is reflected by the multiple mechanisms that distort PPAR*γ* signaling in cancer. PPAR*γ*-RXR heterodimer binds to specific response elements contained within upstream, intronic, and downstream sequences of target genes. The ability of this heterodimer to participate in either transactivation or transrepression is disrupted by multiple mechanisms in cancer cells. (1) *Genetic mechanisms*; although relatively rare, mutations to the PPARG gene occur, as do cytogenetic rearrangements, notably in thyroid cancer with the generation of the PAX-8-PPAR*γ* fusion product. (2) *Epigenetic mechanisms*; the PPAR*γ* receptor normally exists in a dynamic equilibrium with each of two large complexes, namely, coactivator (CoA) and corepressor (CoR) complexes to regulate genes targets. Central components of these complexes are a cohort of ancillary proteins that act to regulate a cohort of posttranslational modifications (PTMs) to histone tails and thereby determine local chromatin organization. In cancer, the stochiometry of this equilibrium is disrupted with downregulation of CoA components such as PGC1-*α* and upregulation of CoR components such as NCOR1. The net result is the distortion of gene regulation abilities, most likely in a promoter specific manner. (3) *Posttranslational mechanisms*; PPAR*γ* is regulated by a number of posttranslational modification including sumoylation, which can allow the liganded receptor to retain associations with the CoR complex and bring about ligand-dependent transrepression. The enzymes responsible for this activity appear altered in malignancy suggesting that the levels of sumoylated PPAR*γ* are in turn distorted. In parallel, associated cofactors, such as PBP/Med1, are also regulated by PTMs and further manipulate and PPAR*γ* signaling. (4) *Nuclear receptor network dynamics*; the PPAR*γ* is a member of a highly interactive network of receptors and in malignancy these interactions appear distorted. For example, the ER*α* (E) homodimer is able to repress the PPARG promoter, and equally PPAR*γ* is both coexpressed with, and regulates expression of other receptors such as PPAR*α*, LXRs, FXR, and VDR to coordinate transcriptional programs. (5) *Ligand generation*; PPAR*γ* senses a wide panel of lipophilic ligands many of which are derived from and catabolized downstream of metabolism of arachidonic acid. Key steps include generation of fatty acids, which are PPAR*γ* ligands, through lipooxygenase (LO) activity (e.g., 5-LO). To counterbalance these activities, the generation of prostaglandins is mediated in large part through the actions of cyclooxygenase (COX) activity (e.g., COX-2). While this can also give rise to PPAR*γ* ligands, these effects are protected further by the clearance of potent prostaglandin PPAR*γ* ligands by the actions of enzymes, such as AKR1C3. In malignancy, an inversion of COX-2 to 5-LO occurs, and further protection from generation of potent prostaglandin ligands occurs, for example, through upregulation of AKR1C3. (6) *Dominant transcriptional programs*; the actions of the PPAR*γ* appear to be distorted as a consequence of deregulated dominant transcriptional programs, such as Wnt signaling. These effects are mediated by enhanced *β*-catenin (*β*) levels and include sequestration of PPAR*γ* to *β*-catenin responsive genomic regions. Implicit within this is that there is a high degree of plasticity of PPAR*γ* signaling and that transcriptional signals can be placed within a quantifiable hierarchy.

Genetically, the PPAR*γ* generally appears to retain its integrity. Rare mutations have been reported and more recently dominant negative variants of the receptor were identified although the biological impact remains to be established firmly [179]. Similarly, altered isoforms may be overexpressed in cancer [180–183]. Cytogenetic rearrangement has been identified in follicular thyroid cancer fusing the PAX-8 transcription factor to PPAR*γ*. In vitro studies suggest PAX-8-PPAR*γ* acts in a dominant negative fashion toward wild-type PPAR*γ* [184] (Figure 2).

In parallel to these genetic changes, the actions of PPAR*γ* appear to be attenuated by changes in receptor expression and known cofactors. The range of interactions with partner proteins of PPAR*γ* appears to be altered. Interactions with PGC1-*α* are reduced in several cancers [21, 185, 186]; and oppositely the known CoRs associated with PPAR*γ* are overexpressed and the transcriptional actions of PPAR*γ* are repressed by epigenetic mechanisms involving HDAC3 [187–189]. Equally, the control of posttranslational modifications appears to be altered. *SUSP-1* [64], which removes the SUMO mark (required for ligand-dependent transrepression) appears to be downregulated in a number of breast and prostate cancers [190]. Within the NR network, PPAR*γ* is coexpressed and interacts both positively and negatively with a cohort of other receptors. For example, the ER*α* and Cyclin D1, (itself a well-known ER*α* target gene and CoA) can both repress the PPAR*γ* gene promoter [191, 192].

The natural ligands for PPAR*γ* are diverse and it is more challenging to make definitive statements concerning their altered generation in malignancy. Equally, the ability for PPAR*γ* to act in a significant and ligand-independent manner also reduces, to an extent, the significance of ligand levels. These considerations aside, the patterns of ligand generation for PPAR*γ* appear to be altered in malignancy. The balance between LO and COX-2 is dysregualted to favor generation of PGH production [193] and accompanied by downregulation of PPAR*γ* [194]. This causes an elevation of PGH_2_, which in turn is converted to protumorigenic prostaglandins, such as PGE_2_, through other synthases. The levels of PGD_2_, which gives rise to 15-PGJ_2_, are closely regulated by an aldo-ketoredcutase (AKR1C3) that is upregulated in malignancy [195–199].

An emergent area of distortion is the extent to which PPAR*γ* signaling is at the mercy of more dominant signal transduction and transcriptional programs. The two tumor promotion models associated with signaling by PPAR*γ* involved elevated levels of signaling by the Wnt pathway. These findings combined with observations on the diversity of genes regulated by the receptor suggest that PPAR*γ* signaling displays plasticity in terms of exact promoter choice. Gene regulatory options are distilled by the combination of receptor-associating cofactors and other signal transduction events. For example, overwhelming Wnt signaling pulls *Ppar* *γ* to *β*-catenin gene targets [146]. This plasticity of signaling is probably reflected by the fact that complete loss or mutation of PPAR*γ* in malignancy is relatively rare. Rather, expression is retained but probably sequestrated and distorted by more dominant signaling events. Resolving these interactions will require a quantitative and hierarchical understanding of the signaling paths through which PPAR*γ* combines with other NRs and signal transduction events to regulate cell fates.

## 4. IS PPAR*γ* A LIGAND-ACTIVATED TUMOR SUPPRESSOR?

A tumor suppressor can be characterized as a protein that reduces the probability that a cell in a metazoan will undergo transformation. Initiation and progression of cancer are associated with attenuation, corruption, expression, and protein function of tumor suppressor genes, increasing the likelihood of tumor formation.

Approximately 10 years have past since the first few reports of PPAR*γ* exerting anticancer cellular effects [109, 111]. Taken together the overwhelming body of data suggests that PPAR*γ* can behave as a ligand-activated tumor suppressor.

1. PPAR*γ* ligands through activating PPAR*γ* can inhibit proliferation and induce differentiation and apoptosis of a wide range of neoplastic cell types in vitro and in murine xenograft tumor models.
2. *Ppar* *γ* ^−/−^ mice are more susceptible than wild-type mice to mammary, colon, ovarian, and skin tumors after exposure to carcinogens and enhance tumor formation in some genetic models of cancer, for example, APC_*min*_ model of colon cancer.
3. The actions of these receptors are attenuated in malignancy by genetic, cytogenetic, and epigenetic mechanisms, and ligand generation is compromised.

Set against, these data are two findings of enhanced tumor formation related to PPAR*γ* in murine cancer models. TZD enhances tumor formation in the APC_*min*_ model [141] and the bigenic mice overexpressing PPAR*γ* in the MMTV-neu breast cancer model have more, highly differentiated tumors [157]. In retrospect, these high-profile studies perhaps reveal important facts of the dominant relationship between Wnt signaling over PPAR*γ* in the mouse. This understanding may have important implications for the necessary molecular diagnostics required to target PPAR*γ* therapies most effectively.

## 5. FUTURE DIRECTIONS

### 5.1. Exploiting dietary understanding from chemoprevention

Recently, the appreciation of the impact of diet on either the initiation or progression of cancer has come significantly to the fore. The World Health Organization has now stated that after smoking diet forms the most preventable cause of cancer. Aspects of these relationships are found in breast, prostate, and colon cancer, where the rate of initiation and progression of disease may be influenced both positively and negatively by the cumulative impact of dietary factors over an individual's lifetime. Beyond the specific micro and macronutrient constituents, the energetic status of an individual is emerging as a risk factor with increased calorific intake and decreased energy expenditure, both contributing deleteriously to cancer initiation and progression (reviewed in [200]).

The NR network has emerged as a systemic sensor of lipid and energetic status [201]. This capacity includes components for sensing carbohydrates [202, 203], cholesterol homeostasis through LXRs and FXR, regulation of metabolic rate through TRs, and sensing of diverse lipids by PPARs. Crosstalk within the superfamily ensures that these sensing and regulatory functions integrate with other receptors such as those for sex steroids. Multiple aspects of these relationships are observed in cancer. For example, fatty acids, such as those present in fish oil and a range of other dietary factors, can activate PPAR*γ* and are associated with *in vivo* prevention of colon cancer in mouse models [165, 204–206] and in human trials in breast cancer [207]. Equally, convergence on PPARs and VDR to regulate IGFBPs and other negative regulatory components of the AKT signaling cascade [208] provides attractive targets for therapeutic intervention.

To exploit this, understanding in either dietary guidelines for the general population or as a chemoprevention strategy for groups defined at risk (e.g., by age or molecular diagnostic) is highly demanding. Despite the significance and potential clinical benefit of these relationships, it remains unclear the critical time frame and dose range when dietary factors may be protective against cancer development, for example, during embryogenesis, childhood, or adult life. By comparison, considerable resources were required to elucidate what is now established as a clear causal relationship between cigarette smoke and lung cancer [209]. There are reasons to be encouraged in targeting PPAR*γ* in a chemoprevention context as studies on the consequences of long-term usage TZDs in diabetes patients have revealed a protective benefit against lung cancer [210].

To address the impact of diet on disease, the emerging field of nutrigenomics aims to dissect the impact of dietary factors on genomic regulation, and thereby physiology and pathophysiology, utilizing a range of postgenomic technologies [211, 212]. This level of integration is emerging. For instance, PPAR*γ* polymorphisms recently have been shown to play a role in determining cancer susceptibility only when patients are above a certain body mass index threshold [213]. Exploitation of such understanding will require modeling of these functions in a network context (reviewed in [214, 215]). Most likely, the application of such rational approaches will resolve the significance of PPARs to mediate anticancer actions of potent dietary factors, such as conjugated linoleic acid [130, 216].

### 5.2. PPAR*γ* and the regulation of cellular energetics

A number of deleterious side effects occur through the use of fatty acids as an energy store, including the generation of reactive oxygen species as a result of lipid peroxidation. The PPAR family combines roles in lipid sensing and utilization with cellular protection against lipid excess. Specifically, PPAR*γ* plays a role in fatty acid uptake and transport (e.g., by adipocytes) and acts to control inflammation that can arise from increased adipocyte differentiation and proliferation (reviewed in [217, 218]). These actions are all altered in malignancy. As proposed by Otto Warburg in the 1930s (and summarized later [219]), cancer cells derive their energy increasingly from anaerobic glycolysis; this concept has received renewed support in recent years [220–222]. The altered energetics of cancer cells are common events, and cancer patients frequently display symptoms which in many ways mimic type II diabetes [223]. Associated with many of these events is an increased propensity for local inflammation.

PPAR*γ* therapeutics have been explored within these separate arenas in different disease settings. That is, to regulate fatty acid metabolism and insulin resistance within the metabolic syndrome, to suppress inflammation, for example, in colitis models [224], and to promote mitotic restraint and induce differentiation within cancer cells. These functions are not separated, but rather all distorted within malignancy. The fact that PPARs, in general, and PPAR*γ* specifically play an integrated regulatory role in these processes suggests that new avenues of exploitation will require a more detailed and quantitative understanding of the contribution of PPAR signaling against a tissue and whole body background of inflammation and altered cellular energetics.

### 5.3. Ongoing questions

The current challenges in PPAR*γ* cancer biology include the following.

1. Determine at which stage PPAR*γ* can influence normal tissue self-renewal.
2. Understand in cancer systems which combination of critical cellular processes to exploit: exert mitotic restraint, induce differentiation, regulate local inflammation, and impact on cellular energetic processes.
3. Define to what extent conformationally restricted synthetic ligands (the so-called SPARMS [225]) can regulate target of these cellular processes through selective cohorts of PPAR*γ* target genes.
4. Identify the mechanisms that attenuate, manipulate, dissociate, and redirect PPAR*γ* signaling in cancer cells and address to what extent the proteins involved in these processes are drugable therapeutic targets.
5. Reveal whether this understanding can be best exploited in the setting of either chemoprevention and/or chemotherapy.
6. Quantify, model, and predict to what extent PPAR*γ* is a nodal point within the NR network and other signal transduction process. Establish hierarchies that place PPAR*γ* specifically, and NRs generally, in the context of other signal processes that collectively maintain homeostasis.

## ABBREVIATIONS

APC: Adenomatous polyposis coli
AR: Androgen receptor
CAR: Constitutive androstane receptor
CDKI: Cyclin-dependent kinase inhibitors
ChIP: Chromatin immunoprecipitation
CoA: Coactivator
CoR: Corepressor
COX: Cyclooxygenase
DMBA: 7,12-dimethylbenz[a] anthracene
ER*α*: Estrogen receptor *α*
FXR: Farnesoid X receptor
GR: Glucocorticoid receptor
HAT: Histone acetyltransferase
HDAC: Histone deacetylase
HETE: Hydroxyeicosatetraenoic acid
HODE: Hydroxyoctadecadienoic acid
IGFBP: Insulin-like growth factor binding protein
LXR: Liver X receptor
NCOA/SRC: nNuclear receptor coactivator/steroid receptor coactivator
NCOR1: Nuclear corepressor
NR: Nuclear receptor
P: Prostaglandin
PBP/MED1: PPAR*γ* binding protein/mediator 1
PGC1-*α*: Peroxisome proliferator-activated receptor *γ* coactivator 1 *α*
PPAR*γ*: Peroxisome proliferator-activated receptor *γ*
PSA: Prostate specific antigen
RAR: Retinoic acid receptor

RXR: Retinoid X receptor
SIRT: Sirtuin 1
SUMO: Small ubiquitin-related modifier
TCF: T-cell factor
TNF: Tumor necrosis factor
TR: Thyroid receptor
TZD: Thiazolidinedione
VDR: Vitamin D receptor
15d-PGJ_2_: 15-deoxy-^12,14^-prostaglandin J_2_.
